# A multicentre, randomized, controlled open-label trial to compare an Accelerated Rule-Out protocol using combined prehospital copeptin and in-hospital high sensitive troponin with standard rule-out in patients suspected of acute Myocardial Infarction – the AROMI trial

**DOI:** 10.1186/s13063-018-2990-z

**Published:** 2018-12-12

**Authors:** Claus Kjær Pedersen, Carsten Stengaard, Hanne Søndergaard, Karen Kaae Dodt, Jakob Hjort, Morten Thingemann Bøtker, Christian Juhl Terkelsen

**Affiliations:** 1 Department of Cardiology, Aarhus University Hospital, Palle Juul-Jensens Boulevard 99, 8200 Aarhus N, Denmark; 2 Department of Cardiology, Regional Hospital Central Jutland, Heibergs Allé 4, 8800 Viborg, Denmark; 3 Department of Internal Medicine, The Regional Hospital in Horsens, Sundvej 30, 8700 Horsens, Denmark; 4 Department of Clinical Medicine, Aarhus University, Aarhus, Denmark; 5 Research and Development, Prehospital Emergency Medical Services, Central Denmark Region, Olof Palmes Allé 34, 8200 Aarhus N, Denmark

**Keywords:** AMI, ACS, Rule-out, Copeptin, High-sensitivity troponin, hs-cTn, Length of stay, LOS

## Abstract

**Background:**

Suspicion of acute myocardial infarction (AMI) is among the most common reasons for admission to hospital in Denmark. Owing to this suspicion, an estimated 50,000 patients are admitted every year. Only 15–20% are finally diagnosed with AMI, whereas 40% are discharged after rule-out of AMI and without initiation of any treatment or need for further admission. In patients discharged after rule-out, the current diagnostic protocol, using consecutive troponin measurements, results in an average length of stay (LOS) of 8–12 h. This leads to overcrowding in both the emergency departments and coronary care units. Measuring copeptin and high-sensitivity cardiac troponin (hs-cTn) upon hospital arrival has shown potential for early rule-out of AMI. However, the diagnostic performance may be improved by accelerating the copeptin measurement of blood sampled already in the pre-hospital phase. Additional evidence on LOS reduction and safety of the rule-out strategy in a large cohort of all-comers is needed.

**Methods/design:**

The rule-out potential is being evaluated in a randomized controlled trial including 4800 patients admitted to hospital for suspicion of AMI. Patients are randomized to either standard rule-out (consecutive troponin measurements) or accelerated rule-out (copeptin measured in a blood sample acquired before hospital admission, combined with troponin measured in the first blood sample upon admission).

**Discussion:**

Sampling blood for copeptin analysis already in the pre-hospital phase and combining this with a later hs-cTn measurement may be the optimal timing for achieving the best diagnostic performance in an AMI rule-out protocol/strategy. Moreover, we are directly comparing pre-hospital and in-hospital blood sample results to address this issue of timing, and we also are comparing single-marker strategies with dual-marker strategies. If the combination of copeptin and hs-cTn is confirmed to rule out AMI safely, implementation of this fast rule-out protocol could optimize patient flow, reduce health care expenses and enable allocation of resources to patients with confirmed illness. In future, when point-of-care analyses of copeptin and hs-cTn are available, hospitalization of the large proportion of patients with symptoms raising suspicion of AMI could potentially be avoided.

**Trial registration:**

ClinicalTrials.gov, NCT02666326. Registered on January 24, 2016.

**Electronic supplementary material:**

The online version of this article (10.1186/s13063-018-2990-z) contains supplementary material, which is available to authorized users.

This protocol covers the full study according to Standard Protocol Items: Recommendations for Interventional Trials (SPIRIT) recommendations (*see* Fig. 1 and SPIRIT checklist in Additional file 1).

**Fig. 1:**
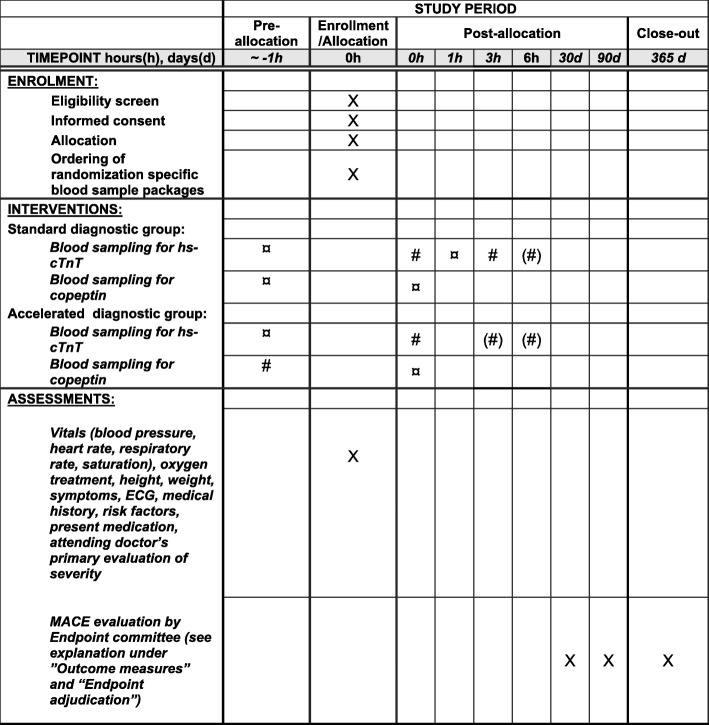
Standard Protocol Items: Recommendations for Interventional Trials (SPIRIT) diagram. ^#^ Result of blood sample analysis is reported to the clinical personnel. ^¤^ No result of blood sample analysis is reported to the clinical personnel. ^(#)^ Performed depending on result of prior samples

## Background

Suspected acute myocardial infarction (AMI) constitutes one of the largest patient groups in emergency medicine, accounting for 6% of all acute health care contacts and up to 27% of medical admissions [1]. In Denmark, more than 50,000 people are hospitalized annually with suspected AMI [2]. Emergency departments (EDs) and coronary care units (CCUs) are struggling with overcrowding, and patients with suspected AMI represent a huge health care challenge [1, 3]. The majority of these patients suspected of AMI have reasons for chest discomfort other than AMI, and most are discharged without any initiation of treatment [2]. The suspicion of AMI dictates immediate emergency medical services (EMS) dispatch, emergency hospital admission, cardiac surveillance and consecutive blood sampling [4]. Thus, a huge potential lies in optimizing patient management by earlier differentiation of patients with versus without AMI.

According to the Universal Definition of Myocardial Infarction, the diagnosis of AMI requires demonstration of a rise or fall pattern of cardiac biomarkers in the blood, with at least one value above the 99th percentile [5]. Therefore, consecutive measurements of two cardiac troponin (cTn) samples at intervals of 3–6 h is the present gold standard procedure to confirm or rule out AMI [4].

The standard diagnostic procedure often results in prolonged admissions for the patients in whom AMI is ruled out, because a final decision may not be reached until results of all analyses are available. Even when a high-sensitivity cardiac troponin (hs-cTn) assay is used, the length of stay (LOS) is between 8 and 12 h for patients discharged after rule-out of AMI [6, 7]. The health care-related costs of these admissions are significant [8].

**Fig. 2:**
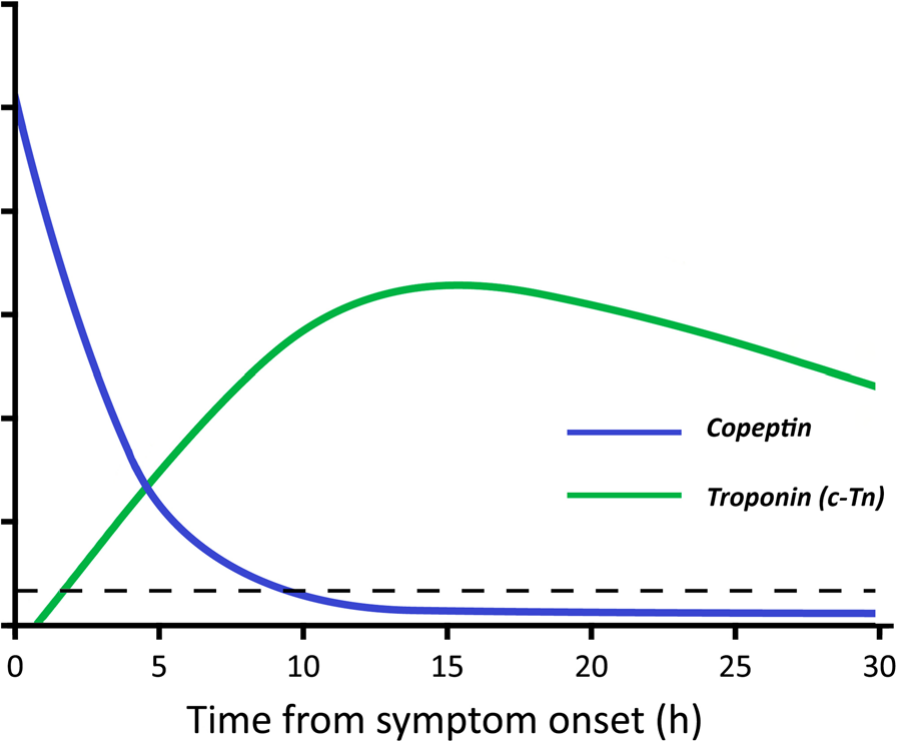
Temporal release kinetics of troponin and copeptin in acute myocardial infarction. Release pattern of copeptin versus conventional biomarkers in the first 24 h since symptom onset. Reproduced with permission from reference [19]

Copeptin has been suggested as an additional biomarker for diagnosing AMI [9–15]. Copeptin is a by-product of the production of arginine vasopressin (AVP), which is also known as antidiuretic hormone. AVP is part of the human endogenous stress response. It is released immediately from the pituitary gland as part of the humoral response to severe stress, including an AMI. AVP is difficult to measure and has a short half-life in blood, but copeptin is stable and easy to measure [16, 17]. In case of an AMI, copeptin is released early, reaches peak concentrations in the blood within the first hours after onset of symptoms, and returns to normal levels within 4–12 h. Thus, the release kinetics are inverse to those of cTn (*see* Fig. 2) [12, 17–19].

Previous studies have suggested using the combination of copeptin and high-sensitivity cardiac troponin T (hs-cTnT) for fast and reliable rule-out of AMI [7, 18]. However, in the one randomized study performed so far, sample sizes were small, and the event rates were low in those where AMI was ruled out [7]. This leads to doubt about the safety of using the hs-cTnT/copeptin strategy rather than serial hs-cTnT measurements. Furthermore, previous studies use a strategy whereby copeptin is measured in a blood sample acquired upon arrival at hospital. Considering the very early release of copeptin, measuring the biomarker as close to symptom onset as possible, preferably before the patient reaches hospital, improves the negative predictive values [20]. In contrast, troponin is ideally measured late after symptom onset owing to its late release from the damaged myocardium [18]. Results from the PreHAP (Pre-hospital diagnosis and triage of heart attack patients) trial demonstrated a potential for safe rule-out of AMI by the combination of pre-hospital copeptin and first in-hospital hs-cTnT [20].

No prospective studies have investigated the potential for LOS reduction and the safety of a rule-out strategy with optimal timing of blood sampling which combines pre-hospital copeptin levels with troponin levels measured upon admission. A large randomized controlled trial is needed to examine whether this accelerated rule-out strategy is a safe and reliable tool for rapid rule-out of AMI.

### Objectives

#### Primary objectives

Is early discharge based on the combined biomarker analysis associated with the following:

- Total duration of hospital stay?
- Time to decision regarding discharge or continued hospitalization?

Is early discharge based on the combined biomarker analysis associated with major adverse cardiac events (MACE) (*see* “Primary outcomes measures” section below)?

#### Secondary objectives

1. Is early discharge based on the combined biomarker analysis associated with early re-admission in patients with specific characteristics?
2. Is early discharge based on the combined biomarker analysis associated with a change in patient experience, patient needs during and after admission, and mental health?
3. Is early discharge based on the combined biomarker analysis cost-effective from a public perspective, regarding staff resources, costs of hospital stay, adherence to the labour market, and use of other health care services (cost-benefit analysis)?

#### Tertiary objectives

1. To evaluate the diagnostic performance characteristics of the accelerated rule-out algorithm as compared with standard diagnostics
2. To evaluate the prognostic value of the accelerated rule-out algorithm as compared with standard diagnostics

### Hypothesis

We hypothesize that the combined measurement of pre-hospital copeptin and in-hospital high-sensitivity troponin

1. Reduces admission time by 12.5% in patients in whom AMI is ruled out
2. Reduces the time to decision of discharge or continued hospitalization in all patients suspected of AMI
3. Is non-inferior compared with the standard rule-out procedure in relation to MACE
4. Is cost-effective from an individual patient perspective and a public health system perspective

## Methods/design

### Aim

We aim to investigate the rule-out potential of copeptin measured in pre-hospital blood samples of patients suspected of AMI in combination with in-hospital hs-cTn compared with the standard rule-out protocol using consecutive in-hospital hs-cTn measurements.

### Study design

The AROMI (Accelerated rule-out of acute myocardial infarction using copeptin and troponin) trial is a randomized, controlled, open-labelled, multicentre trial with two parallel groups and LOS and MACE rate as primary endpoints. MACE rate is evaluated in a non-inferiority design.

### Setting

#### Study setting

The trial includes patients suspected of AMI who are admitted to a cardiac department at three different hospitals in the Central Denmark Region: two regional hospitals (Regional Hospital Central Jutland in Viborg and Regional Hospital in Horsens) and one tertiary highly specialized centre (Aarhus University Hospital in Skejby), which also functions as a regional hospital for its local catchment area. The study catchment area covers approximately 780,000 inhabitants and presumably 7500–8000 admissions/year with suspicion of AMI.

#### Danish health care system

The Danish health care system is governmental, financed by general taxes, free of charge for all patients. [21] The Danish health care system is divided into a primary sector consisting of general practitioners (GPs), private practice specialists, dentists, and physiotherapists and supportive care offered by the municipalities, and a secondary sector covered by public hospitals.

The GPs function as the patients’ primary contact and as gatekeepers to the rest of the health care system. In the Central Denmark Region, out-of-hours contacts are handled by a telephone and consultation service, provided by GPs or specially trained nurses. If needed, the out-of-hours service can refer the patient for hospitalization.

EMS is accessible via the emergency telephone number 1-1-2. EMS response for patients suspected of AMI includes an obligatory first tier of ambulance staffed with emergency medical technicians (EMTs) and/or paramedics and, for patients with respiratory or hemodynamic compromise, a second tier of ground-based, physician-staffed pre-hospital critical care teams or physician-staffed helicopter EMS in case of long-distance missions. All pre-hospital patient handling, diagnosis and treatment follows strict regional standard operating procedures.

Depending on the suspected condition, patients are transported to either 1 of the 21 EDs or to relevant specialized departments. Patients self-presenting at hospitals are in general rare in Denmark, probably owing to the high availability of primary-level care and EMS.

#### Admission and pre-hospital management of patients suspected of AMI in the Central Denmark Region

When suspicion of AMI is raised (by GP, out-of-hours service or EMS dispatch centre), patients are referred for hospitalization by ambulance. If the patients’ symptoms fulfil the EMS criteria for suspected AMI (defined as recent or ongoing prolonged symptoms of chest discomfort, new onset of dyspnoea in patients without prior pulmonary disease, or clinical suspicion of AMI), a venous blood sample is drawn for point-of-care (POC) analysis of cTn, and an electrocardiogram (ECG) is sent to the local cardiology department for interpretation. (Telemedicine conference between paramedic and cardiologist is performed via mobile telephone while the patient is still in the ambulance.) The cardiologist on call contacts the ambulance, and a tentative diagnosis is established, taking all available information (patient history, symptoms, transferred ECG and POC troponin) into account. Patients with relevant symptoms and risk factors for ischaemic heart disease or other cardiac causes are triaged directly to a department of cardiology for rule-in or rule-out of AMI or other relevant diagnostics and treatment. Patients with ST-elevation myocardial infarction (STEMI) or suspected high-risk cardiac conditions (including patients suspected of being at high risk of AMI, defined as patients with clear, characteristic symptoms of AMI and either significant ECG changes or elevated POC troponin) are triaged to a percutaneous coronary intervention (PCI)-capable cardiac centre. The remaining patients with miscellaneous symptoms and no risk factors are triaged to the ED.

Pre-hospital blood sample collection in patients suspected of AMI is a standard procedure in the Central Denmark Region. This provides a unique possibility to collect two separate timely blood samples at the time of admission.

### Participants

#### Inclusion criteria

1. Patients admitted by ambulance to a CCU on the basis of suspicion of AMI after having ECG diagnostics performed via telemedicine
2. A successful pre-hospital blood sampling is required for inclusion.

#### Exclusion criteria

1. Aged younger than 18 years
2. Patients in whom a firm and valid informed consent is unattainable (e.g., owing to psychiatric disease, dementia, under influence of euphoric substances)
3. Patients triaged directly to the tertiary centre with suspected STEMI or for cardiac reasons other than suspected AMI (ventricular tachycardia, ventricular fibrillation, and third-degree atrioventricular block)
4. Patients who, at the time of admission, are known to have central diabetes insipidus
5. Patients in whom an obvious alternative diagnosis is suspected at the time of arrival to hospital (e.g., new supraventricular tachycardia, pulmonary embolism, aortic dissection) and without suspicion of AMI

At Aarhus University Hospital, patients are included only between 0800 and 2200 on weekdays (no inclusion on weekends or holidays). This is due to organizational procedures at the in-hospital central laboratory, which results in limitation of out-of-hours availability of copeptin analysis.

### Interventions

**Fig. 3:**
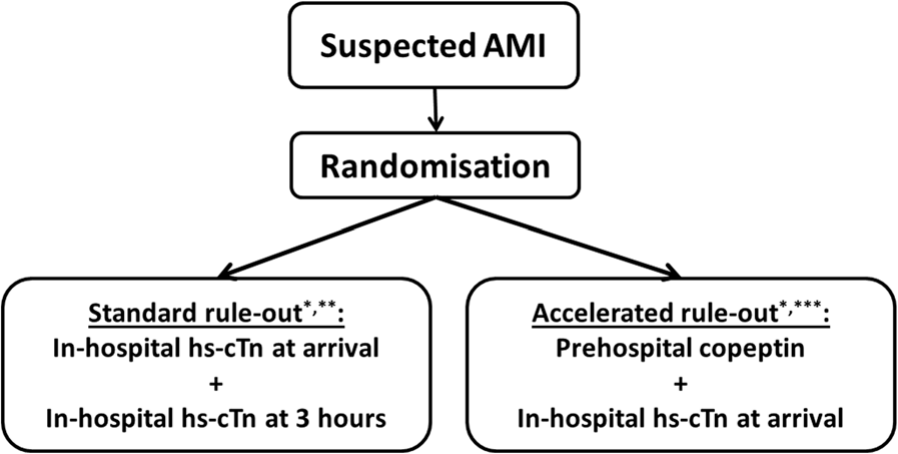
Study flowchart. Flowchart of patient courses in the two randomization groups. * Further diagnostic workup, including additional hs-ctn, is fully at the discretion of the attending physician. ** AMI is ruled out if both hs-ctn measurements are < 99th percentile URL or no significant rise or fall in hs-ctn is seen. *** AMI is ruled out if copeptin is < 95th percentile URL and hs-ctn < 99th percentile URL. *AMI* Acute myocardial infarction, *hs-ctn* High-sensitivity troponin, *URL* Upper reference limit

*See* Fig. 1) for the study SPIRIT figure and Fig. 3) for study flowchart.

#### Intervention groups

Patients are randomized in a 1:1 allocation ratio into either of two diagnostics groups—the standard or accelerated diagnostic group—at the time of arrival to the hospital. “Standard” diagnostics follow the present standard diagnostic algorithm in Denmark, which is the European Society of Cardiology (ESC) 0 h/3 h algorithm. Patients have hs-cTn measured on arrival, depending on the result and the patient’s symptoms; hs-cTn is re-measured 3 h later. If both measurements are below the diagnostic cut point (the 99th percentile of healthy people), AMI is ruled out. The patient may then be discharged or receive further diagnostic workup or treatment for other conditions at the discretion of the attending physician.

The 0-h algorithm is not used in the Central Denmark Region, owing to patients’ difficulty in recalling changes in symptoms within the preceding 6 h. Thus, the majority of patients have at least two hs-cTn measurements performed.

“Accelerated” diagnostics include analysis of copeptin in a blood sample obtained in the pre-hospital phase and analysis of hs-cTn in a single blood sample drawn at the time of arrival at the hospital. All biomarker analyses are performed at the central hospital laboratory because POC instruments for pre-hospital copeptin measurement are not available.

If both makers are below the diagnostic cut-point (the 95th percentile of healthy people for copeptin and the 99th percentile of healthy people for hs-cTn), AMI is ruled out. The patient may be discharged or further diagnostic workup or treatment may be initiated at the discretion of the attending physician.

If either of the two biomarkers is above the mentioned cut points, AMI is not ruled out. Additional testing is decided by the attending physician. Unless alternative diagnoses are obvious, patients will have hs-cTn re-tested 3 h later as per the “standard” rule-out procedure.

#### Modifications

In both randomization groups, additional treatment and diagnostics (including repeat hs-cTn measurement) are fully at the discretion of the attending physician. Patients can withdraw from the study at any time point and will, if still admitted, follow the standard rule-out protocol (but without copeptin measurement, collection of extra blood samples or collection of blood samples for later use).

#### Adherence

All personnel are trained by local supervisors and are continuously reminded: 1) to follow the protocols for each randomization, and 2) regardless of randomization, to make a plan for the patient’s further course, taking the possible blood sample results into account. The written plan in the patient’s medical record could be as follows: ‘If troponin and copeptin/first and second troponin are normal, then…. If not, then…’.

### Concomitant care

#### Pre-hospital and in-hospital blood sampling

In the Central Denmark Region, all patients suspected of AMI have blood sampling performed by EMTs/paramedics in the ambulance before reaching hospital. The blood sample is drawn from a newly introduced peripheral venous catheter before flushing, using a blood collection tube with manually induced vacuum (Sarstedt Lithium-Heparin Coated S-Monovette, 5.5 ml; Sarstedt AG and Co., Nümbrecht, Germany). All tubes are labelled with a patient identifier (ID), date and time of collection. The blood sample is brought with the patient to the hospital, where it is collected by a central laboratory technician. The in-hospital blood samples are collected by the central laboratory technician according to local standard procedures and using standard tubes.

#### Handling of blood samples

The pre-hospital and first in-hospital samples are processed at the central hospital laboratory. Plasma is separated by centrifuging at 2500 *g* for 10 min and aliquoted into two samples for copeptin and hs-cTn analysis. The remainder of the copeptin sample is frozen at − 80 °C for storage in a biobank (*see* Additional file 2). Regardless of the patient’s randomization, the pre-hospital and first in-hospital samples are analysed for both hs-cTn and copeptin, but only results relevant to the assigned randomization are reported to the clinicians (*see* Fig. 1). Besides hs-cTnT and copeptin, a series of other analyses (e.g., white blood cell count, haemoglobin, lipid status, P-natrium, P-potassium, according to local procedures) are performed using the first in-hospital blood sample.

#### Additional in-hospital blood samples

Patients randomized to the standard diagnostic group will have a second (third if you count in the extra study sample; *see below*) blood sample taken after 3–6 h (*see below*). Patients randomized to accelerated diagnostics will only have a second blood sample drawn if copeptin, troponin or both are elevated in the initial blood samples or if requested by the attending physician. Second and later hs-cTn samples and analysis are performed as described above, except that copeptin is not analysed, and the remaining plasma is not stored. As a supplement, all patients in the standard diagnostic group will have an extra blood sample drawn 1 h after the first in-hospital blood sample. This sample is only analysed for hs-cTn, and no blood is stored. The extra sample is not drawn in the accelerated diagnostic group, because some of these patients potentially could be discharged at or before this time point.

#### Sample request and reporting

Blood samples are requested by the personnel at the cardiac department according to the patient’s randomization status (for randomization process; *see below*). Requests for blood sample analysis are done by ordering pre-defined bundles/packages of analyses. This ensures that all patients have both copeptin and hs-cTnT measured in both pre-hospital and first in-hospital blood samples, but only results relevant to the randomization group are passed on to the clinicians, as follows:

- No copeptin results and no result of the 1-h troponin in the standard group
- Results of pre-hospital but not in-hospital copeptin in the accelerated diagnostic group

All hs-cTn analyses are ordered with prioritization, meaning that the results have to be reported within 1 h.

Results are reported electronically from the hospital laboratory system (LABKA II; CSC Healthcare EMEA/DXC Technology, Boeblingen, Germany) to the clinicians via the electronic patient management system (Midt-EPJ; Systematic, Aarhus C, Denmark). All blood sample analysis results (including the blinded pre-hospital copeptin in the standard group and the in-hospital copeptin) can be retrieved from LABKA on request.

#### General in-hospital patient management

The patients are received by a doctor and a nurse at the department of cardiology, and the patient is immediately put on continuous ECG surveillance. The initial assessment includes ECG, vital signs, thorough questioning of medical history and present symptoms, and a physical examination. A laboratory technician is called for prompt blood sampling. All blood sample analyses are performed at a central hospital laboratory. Acute echocardiography and additional diagnostic procedures such as imaging, stress testing and additional biochemistry are available at all centres. Supplementary standard diagnostic workup for each centre is described below:

- Aarhus: Echocardiography is performed as standard in connection with first evaluation by the attending physician. Chest x-ray is performed on demand according to the clinical presentation. First and second evaluations (including follow-up of initial blood sample analyses) are often performed by the same physician.
- Viborg: Echocardiography is performed as standard in connection with the secondary evaluation. Chest x-ray is performed on demand according to the clinical presentation. Patients in Viborg are generally followed during evening rounds by a senior physician or on the general rounds the next day.
- Horsens: Echocardiography is performed according to the clinical presentation. Chest x-ray is performed as standard on all patients. First and secondary evaluations (including follow-up of initial blood sample analyses) are often performed by the same physician.

### Outcomes

#### Outcome measures

##### Primary outcome measures

1. LOS:
  - Time (hours and minutes) from admission to discharge from cardiac department (either to home or to a non-cardiac department)
  - Retrieved from the patient administrative system, the patient’s medical record and the national health registry
  - Will be evaluated when 300 patients have been discharged after rule-out of AMI at each site (patients discharged from hospital within 12 h of admission)
2. Combined MACE proportion
  Combined endpoint of MACE (only first event per patient), consisting of the following (Occurring within time from randomization to 30 days after randomization):
  All-cause mortality
  Survived cardiac arrest
  Confirmed acute coronary syndrome (ACS) or re-admission with ACS
  Non-scheduled coronary intervention
  Life-threatening arrhythmias
  Will be evaluated when inclusion of all patients is completed

(*See below* for detailed description of each component of the combined endpoint.)

##### Secondary outcome measures

ᅟ

##### Combined MACE proportion

- As described above within 90 and 365 days after randomization
- Will be evaluated when inclusion of all patients is completed

##### All-cause mortality

- Number of all-cause deaths divided by number of patients
- All-cause mortality registered in the national health registry, occurring from time of admission to discharge, within 30, 90 or 365 days after randomization
- Will be evaluated when inclusion of all patients is completed

##### Cardiovascular mortality

- Number of cardiovascular deaths divided by number of patients, occurring from time of admission to discharge, within 30, 90 or 365 days after randomization
- Cause of death is registered in the national register of causes of death
- Will be evaluated when inclusion of all patients is completed

##### Survived cardiac arrest

- Number of patients surviving an event of cardiac arrest divided by number of patients
- “Survived cardiac arrest” is determined from registration of out-of-hospital cardiac arrest in Danish register of cardiac arrest and in-hospital cardiac arrest in DANARREST and in the national health registry, occurring from time of admission to discharge, within 30, 90 or 365 days after randomization
- The endpoint committee adjudicates survived cardiac arrest, blinded to the initial randomization.
- Will be evaluated when inclusion of all patients is completed

##### Confirmed diagnosis of ACS during index admission or re-admission with ACS

- Number of ACS events (only first event per patient) divided by number of patients
- The national health registry is used to determine whether the patient is confirmed of having or being re-admitted with ACS, from time of admission to discharge and within 30, 90 and 365 days after randomization. The endpoint committee adjudicates ACS, blinded to the initial randomization. The 2015 ESC Guidelines for the management of ACS in patients presenting without persistent ST-segment elevation and the Universal Definition of Myocardial Infarction will be used to evaluate if the patient had ACS and subsequently classify it as follows:
  ○ Unstable angina pectoris (UAP)
  ○ Non-ST-elevation myocardial infarction (NSTEMI)STEMI
  ○ Bundle branch block myocardial infarction
- Will be evaluated when inclusion of all patients is completed

##### Non-scheduled coronary intervention

- Number of non-scheduled coronary interventions (only first event per patient) divided by number of patients
- The national health registry is used to determine whether the patient has a non-scheduled intervention performed. Time from index admission to first intervention and type of intervention (PCI or coronary artery bypass graft [CABG]) is determined. The endpoint committee adjudicate interventions blinded to original treatment strategy.
- Will be evaluated when inclusion of all patients is completed

##### Life-threatening arrhythmias

- Number of events with life-threatening arrhythmia (only first event per patient) divided by number of patients
- The national health registry is used to determine whether the patient is diagnosed or re-admitted with a life-threatening arrhythmia, defined as ventricular tachycardia, ventricular fibrillation or third-degree (total) atrioventricular block within index admission and within 30, 90 or 365 days of randomization. The endpoint committee adjudicates re-admission with life-threatening arrhythmia, blinded to the initial randomization.
- Will be evaluated when inclusion of all patients is completed

##### Other outcome measures

ᅟ

##### Diagnostic performance characteristics of the accelerated rule-out algorithm as compared with standard diagnostics

- Sensitivity, specificity, positive predictive value and negative predictive value for the diagnosis of AMI will be evaluated in interim analysis after 300 patients have been discharged after rule-out of AMI in each site (patients discharged from hospital within 12 h of admission)

##### Prognostic value of the accelerated rule-out algorithm as compared with standard diagnostics

- Mortality within 1, 7, 30 and 365 days
- Will be evaluated in interim analysis after 300 patients have been discharged after rule-out of AMI in each site (patients discharged from hospital within 12 h of admission)

##### Cost efficiency

- Costs and cost-effectiveness will be evaluated and compared between the two diagnostic strategies.
- Will be evaluated in interim analysis after 300 patients have been discharged after rule-out of AMI in each site (patients discharged from hospital within 12 h of admission)

##### Risk factors and patient experiences

- We plan to evaluate the patients’ experience of being discharged earlier and risk factors for early re-admission.

##### Effect of the accelerated model on each outcome component in the primary outcome

- Regression models of each outcome component, including randomization group, age, sex, symptoms, time from onset of symptoms, known co-morbidities, pre-existing ischaemic heart disease, ECG changes, site of inclusion, season, and time since trial start-up

#### Participant timeline

*See* Fig. 1. Patients are enrolled as early as possible after admission. Procedures of importance for acute patient safety will precede the inclusion process. The attending nurse will inform and, if eligible for inclusion, enrol and randomize the patient when relevant acute procedures are completed or initiated. The attending nurse and physician collect baseline information (*see* “Data collection methods” Section).

Patients assigned to the standard group are scheduled to have two consecutive in-hospital hs-cTn measurements with an interval of 3 h. Adhering to the study protocol will result in a minimum admission time of 4 h: initial assessment/treatment + enrolment, allocation and first blood sampling + 3-h interval to second sampling + laboratory turnaround time. Additional treatment and diagnostic procedures (including repeat hs-cTn measurement) are fully at the discretion of the attending physician.

Patients assigned to the accelerated group are scheduled to have one in-hospital hs-cTn measurement performed, which will result in a minimum admission time of 1 h: initial assessment/treatment + enrolment, allocation and blood sampling + laboratory turnaround time. Additional treatment and diagnostics (including repeat hs-cTn measurement) are fully at the discretion of the attending physician. The endpoint committee will assess MACE events within 30, 90 and 365 days (*see* “Data collection methods” section).

### Sample size

The total sample size will be 4800 patients.

#### Sample size calculation

Thirty-day MACE including all-course mortality is expected to be between 5.5% and 7.5% [7, 20]. Using a MACE rate of 6.5%, a non-inferiority limit of 2%, alpha = 2.5% and power (beta) = 80% will result in the need for enrolment of 4772 patients to confirm a difference in 30-day MACE rate of more than 2%.

Regarding LOS, in 2012, the average LOS of patients admitted with suspected AMI at Aarhus University Hospital was 12 h (SD = 6.1). It is expected that 40% of patients in the accelerated group can be discharged within 3 h after admission [7, 20], thus resulting in an estimated 1–1.5 h reduction of LOS in patients discharged without AMI.

Inclusion of about 4800 patients in total will enable us to show a difference in LOS of 54 min with a power > 90%. To show an average 1.5-h reduction in LOS (under the condition that 40% in the accelerated group are discharged within 3 h), 337 patients in each randomization group discharged after early rule-out of AMI (patients discharged from hospital within 12 h of admission) are needed (μ = 12, SD = 6.1, alpha = 5%, beta = 90%). To take differences between sites into account, we increased the number of discharged patients by one-third to 450 patients per randomization group. Distributing the patients equally between sites (150/site/randomization group), 300 patients discharged after rule-out of AMI (patients discharged from hospital within 12 h of admission) in each site are needed.

### Recruitment

All study sites have local study representatives and receive regular visits and contacts from the principal investigator (PI) to ensure high local engagement and recruitment. All nurses at the participating departments are trained to perform enrolment of patients.

### Informed consent and assignment of interventions

#### Consent

Patients are enrolled by the attending nurse at presentation. All nurses at the participating departments are trained to perform enrolment of patients.

Information, collection of informed consent, and randomization/allocation are done using the TrialPartner web-based trial system. TrialPartner is accessible from a tablet and has all study information available to support patient information, including the full written information and a study description for study personnel.

The attending nurses will do the following at the time of admission:

- Inform the patients in both oral and written form
- Collect signed informed consent to participate in the study
- Enter site, personnel ID, patients’ wishes regarding information of results, and patients’ diabetes status (yes/no)
- Press the randomization–button to determine allocation (*see* “Allocation” section below).

#### Additional studies

In parallel, the nurses will also collect signed informed consent forms from the patients for storage of remaining blood plasma in a biobank (*see* Additional file 2).

#### Allocation

Web-based computer randomization is used to allocate patients to the treatment groups at a 1:1 ratio, stratified by site and diabetes status (yes/no), by the method of permuted block randomization with random varying block sizes. The trial system (TrialPartner) permits, with a personal log-in, 24-h randomization. All actions in the system are logged. Patients are randomized and allocated using a web-based solution from a tablet. The allocator needs to enter the patient’s social security number; present written information; retrieve the patient’s signature on consent (via the tablet); sign the consent (via the tablet); and input site, diabetes status (yes/no) and ID before the web database will allow allocation.

The allocation sequence is generated by a local data manager at Aarhus University and incorporated into the web-based TrialPartner. All information on allocation sequence is blinded to study personnel, including the authors. After allocation, the nurse or other personnel at the department order blood sample packages according to allocation (*see* “Sample request and reporting” section).

### Blinding

The AROMI study is by design an open, unblinded study because disclosure of the assigned group and associated blood sampling should enable an altered patient management. Members of the endpoint committee will be blinded to allocation. Because the committee will have full access to patient file information from the cardiac departments, there will be cases where the allocation will be revealed or appear obvious to the committee members.

### Data collection, management and analysis

#### Data collection methods

Raw data are collected from several sources, outlined below.

##### Case report form

The attending personnel complete a case report form (CRF) in which baseline characteristics, symptoms and ECG at presentation, clinical evaluation, time of admission and time of discharge, presumed MACE during admission, and diagnostic procedures during admission are documented.

##### Patient medical record

Missing or non-reported data regarding baseline characteristics, symptoms and ECG at presentation, and diagnostic procedures are supplemented from the medical record. To ensure uniformity, the supplementation is performed by three trained assessors. Medical records are also used for identifying suspected MACE events (*see* “Adjudication of events” section).

##### Laboratory information management system

Data on blood sampling, including date and time of blood sampling, name and Nomenclature for Properties and Units code of the analyses, and results are retrieved from the laboratory information management system (LABKA).

##### Registries

Data are retrieved from several registries. Registry data are used for supplementing missing or non-reported data regarding baseline characteristics and for identifying suspected MACE events (*see* “Adjudication of events” section). The list of registries and registry data used can be found in Additional file 3.

#### Endpoint committee adjudication

The endpoint committee will adjudicate MACE endpoints and report these electronically to the PI (*see* “Adjudication of events” section).

#### Data material

We will collect data on the following groups of measures (*see* Additional file 3 for further specification):

Outcome data:

- LOS: derived from time of admission and time of discharge
- MACE events (*see* Adjudication of events)

#### Baseline characteristics

- Vital signs
- Symptoms
- ECG

Attending doctor’s evaluation of severity after initial examination (doctor’s “gut feeling”): dichotomous: serious condition or not serious condition

- Timing (symptoms, admission)
- Presumed MACE during and after index admission
- Diagnostic procedures during and after index admission
- Biochemistry results during and after index admission
- Interventions performed during and after index admission
- Additional admissions after index admission

### Adjudication of major adverse cardiac events

The components of the primary outcome (MACE endpoints and time of MACE endpoints) are adjudicated via a combination of auto-adjudication and manual adjudication.

#### Auto-adjudication

A subset of the events are so extensively documented in the mentioned registries and databases that an automatic adjudication has been accepted by the endpoint committee. To further validate this procedure, at least 10% of all automatic adjudications are subsequently confirmed by the endpoint committee.

#### Manual adjudication

The manual adjudication is performed by an independent endpoint committee consisting of six to nine (depending on the final number of suspected events; *see below*) Danish independent cardiologists from outside the Central Denmark Region (*see* Additional file 4). Because of the high level of completeness and quality of the Danish health care registries and available electronic databases, a complete review of medical records from all patients is not considered to be necessary. Instead, only selected suspected events are presented to and evaluated by the endpoint committee (*see below*). Each suspected event is independently evaluated by two cardiologists. The adjudication is based on patient medical records, laboratory results and other supplementary information on request from the adjudicators. The endpoint committee is, per protocol, blinded to randomization status; the assigned group may be derived de facto from the medical record in most cases. All other information is available in the adjudication process.

All-cause death is accepted if the patient is registered as dead in the social security register. Survived cardiac arrest is accepted if a resuscitation attempt by a health care professionals is registered in the patient medical record and the patient survived to discharge. Non-scheduled re-vascularizing coronary intervention (PCI or CABG) is accepted if a non-scheduled coronary re-vascularization is registered in the patient medical record. Life-threatening, treatment-demanding arrhythmias are accepted if third-degree atrioventricular block, ventricular tachycardia or ventricular fibrillation resulting in initiation of treatment is registered in the patient medical record. Re-admissions with ACS (including NSTEMI types 1–5, STEMI types 1–5 and UAP) are accepted according to the criteria described in the Universal Definition of AMI (*see* Additional file 4 for full description). Myocardial injury is adjudicated by the endpoint committee but is not included as part of the endpoint of re-admissions with ACS.

The adjudication is documented in TrialPartner by each adjudicator, and the event is confirmed if both adjudicators agree. In case of disagreement between the two reported adjudications, the event is evaluated by a third member of the endpoint committee (*see* Additional file 4).

#### Suspected events

A pre-adjudication is performed before endpoint committee assessment. Suspected events are automatically identified from all available data (*see* “Data materials” section) using STATA15 IC statistical/data management software (StataCorp, College Station, TX, USA).

Suspected events and automatic adjudication are defined for each possible endpoint of MACE. *See* the complete description of suspected events, MACE adjudication and automatic adjudication in Additional file 4.

#### Retention

Because the patients’ part of this study is completed as soon as a diagnosis of AMI is confirmed or rejected during index admission, there is no need for participant retention plans. The Danish health care registries are of high quality and have a high rate of completeness. Data will be analysed on both an intention-to-treat and per-protocol basis to accommodate issues of protocol non-compliance/deviation.

#### Data management

##### Data entry

Only data collected via the paper CRF are entered manually into the electronic case report form (in TrialPartner). This entry is done by a limited number of trained members of the study staff. All other data are collected from registries using the participant’s unique social security number for identification.

##### Data quality

To minimize mistakes during data entry, TrialPartner has an incorporated live range check, giving warnings if data are outside of limits. Entered data are double-checked by the regional good clinical practice unit (GCP) for 10% of the entered data. Moreover, several of the variables are retrieved from more than one source, compared and corrected in cases with errors.

### Confidentiality

All study-related information will, until collection, be stored securely at the study sites in areas with limited access. After collection and entry, laboratory specimens, reports and data collection forms will be identified by a coded participant ID number to maintain participant confidentiality. Forms and any other listings that link participant ID numbers to other identifying information will be stored separately from study records with the participant ID number. No published results or data will include individuals’ personally identifying information.

#### Data storage and security

All manual data are stored safely in a secured designated room with limited access, access control and access registration located at Aarhus University Hospital. Electronic data are stored in a minimum of two copies in encrypted hard drives or secure online servers. All local and server databases will be secured with password-protected access systems, access logging and encryption. Members of the endpoint committee have access to the patient information via encrypted server access and encrypted hard drives.

### Statistical methods

#### Statistical analysis

LOS will be assessed and compared separately for the accelerated vs the standard group and for the rule-out (patients discharged from hospital within 12 h of admission) subgroups of the accelerated group vs the standard group. MACE rate at 30, 90 and 365 days will be estimated and compared between the accelerated and standard diagnostic groups.

Categorical variables will be compared using Pearson’s chi-squared test or Fisher’s exact test as appropriate. Continuous variables are compared using Student’s *t* test (if normally distributed data) or Wilcoxon’s rank-sum test (if non-normally distributed data). Demographic and baseline characteristics will be presented and compared. All statistical analyses are performed as intention-to-treat analyses. A two-sided *p* value greater than 0.05 is considered significant. In non-inferiority analysis a one sided *p* value greater than 0.025 is considered significant. Multiple imputations will be used to handle missing data.

For evaluation of diagnostic properties and prognostic value of the accelerated rule-out algorithm, randomization is ignored and all data are pooled. In these analyses, the authors will be completely blinded to randomization because these analyses include elements of MACE and will be evaluated before full inclusion.

Diagnostic properties will be calculated and presented as sensitivity, specificity, positive predictive value and negative predictive value. These will be calculated for pre-defined subgroups: early presenters (≤ 3 h from onset of symptoms) vs late presenters, young vs middle-aged vs old, no prior vs prior coronary artery disease, gender, prior vs no prior congestive heart disease, groups of symptoms (chest pain [angina or atypical], dyspnoea, stomach pain, other).

Kaplan-Meier cumulative mortality curves, stratified by combinations of AMI/no AMI and hs-cTn and copeptin values above or below the designated cut points will be presented to describe prognostic value. Comparisons between groups are done using the log-rank test. Statistical software (Stata version 15; StataCorp) is used for statistical analysis.

### Monitoring

#### Data monitoring

##### Formal committee

The study is monitored by a data monitoring committee (DMC).

##### Role

The DMC will perform interim analyses to evaluate safety endpoints and study progression. For more information on the DMC, *see* Additional file 5.

#### Harms

The potential harm of the study intervention (early discharge based on copeptin and hs-cTnT) is that patients with an AMI or other serious condition are discharged without having their actual condition detected and treated. If early discharge without relevant diagnosis should occur, we expect to be able to identify this because it is expected to lead to an early re-admission or registration of MACE.

The clinical personnel are instructed to report any case of re-admission with a suspected missed diagnosis to the sponsor/project management. We also evaluate all re-admissions within the first 24 h after discharge from index admission with the DMC (*see* Additional file 5).

#### Auditing

The study is monitored by the local GCP unit. The study does not include the use of medical products in humans. However, we have designed and conducted the study according to GCP guidelines, with some adaptations to apply the setting with a diagnostic protocol as an intervention.

The GCP unit has audited the study initiation. Additionally, data collection and registration are continuously audited, and final data will be audited after the last patient is enrolled.

## Discussion

Early rule-in/rule-out of AMI has been a hot subject for the past 5–10 years. Although patients suspected of AMI are abundant, only about one-tenth of them actually have had an AMI. Biomarker testing is a keystone in AMI diagnostics. Because no diagnostic strategy can rely entirely on biomarker testing, however, biomarker testing should always be performed in parallel with a thorough clinical evaluation.

Several new strategies to improve and accelerate AMI diagnostics have been suggested. These include troponin-only strategies optimizing the ESC standard algorithm for the diagnosis of NSTEMI (0 h/1 h, 0 h/2 h and 0 h algorithms), strategies combining troponin measurement with clinical data (Manchester Acute Coronary Syndromes decision rule, heart score) and dual-marker strategies combining troponin with other biomarkers (e.g., copeptin and Heart-type fatty acid-binding protein).

In the troponin-only strategies, the measurements of hs-cTn are advanced timely, either by reducing time between samples (the delta-time) or by reducing the number of blood samplings. All of these are challenged by the delayed release of troponin after a myocardial infarction. Several retrospective studies have shown the troponin-only strategies to be effective and safe, but they have not been evaluated in larger, randomized trials.

A common weakness in all fast-diagnostics strategies is the inherent need to adjust the local clinical setup to fit the fast-track diagnostic approach to fully exploit the potential of these strategies. All relevant in-hospital diagnostics should be easily available and completed with no delay. Health care personnel should be available for clinical evaluation and decision-making at the time when biomarker results and results of other examinations are delivered.

In several retrospective studies the combination of copeptin and cTnT has shown diagnostic properties comparable to or better than troponin-only strategies. In a single randomized controlled trial it even reduced LOS significantly without increasing the MACE rate [7]. In the AROMI trial setup, we optimize the timing of copeptin analysis by advancing the blood sampling of copeptin to the ambulance. Thereby we expect to enhance the efficiency of the diagnostic rule-out. To evaluate this potential effect, we also directly compare pre-hospital and in-hospital blood sample results of both copeptin and hs-cTn to address the issue of timing. Besides its clear advantages in the diagnostic process in AMI, copeptin could, owing to its non-specific nature, have a potential use in the diagnosis or especially the rule-out of other acute conditions, such as aortic dissection and pulmonary embolism.

In the current study, the two primary endpoints are selected to reflect the primary demands from a diagnostic protocol (efficacy and safety):

1. If the accelerated diagnostic protocol does not improve our diagnostic process towards an earlier determination of AMI, then there is no need for implementing it.
2. If indeed the diagnostic process is accelerated and LOS is reduced, the accelerated rule-out process will be acceptable only if it is at least as safe as the current standard procedure.

The components of MACE events are selected because they are the most serious possible consequences of an AMI. All-cause mortality is chosen because we also want to ensure that no other serious conditions are overseen to an extent where it results in the death of the patient.

This study is expected to be the largest randomized trial to evaluate accelerated rule-out protocols in patients suspected of AMI. We consider a non-inferior limit of 2% to be both clinically relevant and to result in a practically achievable sample size. Although a non-inferiority limit of less than 0.5% (approximately equal to a 10% relative change in MACE rate) would be the clinically most relevant limit, a randomized study using this limit would be practically non-feasible because it would need inclusion of more than 75,000 patients.

If the combination of copeptin and hs-cTn is confirmed to rule out AMI safely, implementation of this fast rule-out protocol may optimize patient flow, reduce health care expenses and enable re-allocation of resources to patients with confirmed illness.

Furthermore, if we, by comparing pre-hospital measurement of both copeptin and hs-TnT to in-hospital measurement, can confirm this to be as effective and safe for rule-out of AMI as in-hospital diagnosis, it would seem evident to implement this as a standard pre-hospital procedure. Thus, in a future setting where POC analysis of copeptin and hs-cTn is available, a safe pre-hospital rule-out may in fact be possible, thereby avoiding the hospitalization of a large proportion of patients with symptoms suggestive of AMI.

## Trial status

The study is currently recruiting patients. As of November 1^st^, 2018, 3,560 patients had been included. The first patient was included on January 26, 2016, and inclusion is expected to be completed during 2019.

## Additional files

Additional file 1: SPIRIT checklist. (DOC 127 kb)

Additional file 2: AROMI biobank for future use. (DOCX 22 kb)

Additional file 3: Data variables and registries. Description of the data variables collected in the study and the registries from which some of the variables are retrieved. (DOCX 29 kb)

Additional file 4: Endpoint committee. Description of the endpoint committee and endpoint adjudication. (DOCX 83 kb)

Additional file 5: Data monitoring committee. Description of the data monitoring committee and data monitoring. (DOCX 22 kb)

## Abbreviations

ACS: Acute coronary syndrome
AMI: Acute myocardial infarction
AROMI: Accelerated rule-out of acute myocardial infarction using copeptin and troponin trial
AVP: Arginine vasopressin
CABG: Coronary artery bypass graft
CCU: Coronary care unit
CRF: Case report form
cTn: Cardiac troponin
cTnT: Cardiac troponin T
DMC: Data monitoring committee
ECG: Electrocardiogram
ED: Emergency department
EMS: Emergency medical services
EMT: Emergency medical technician
ESC: European Society of Cardiology
GCP: Good clinical practice
GP: General practitioner
hs-cTn: High-sensitivity cardiac troponin
hs-cTnT: High-sensitivity cardiac troponin T
LOS: Length of stay
MACE: Major adverse cardiac events
NSTEMI: Non-ST-elevation myocardial infarction
PCI: Percutaneous coronary intervention
PI: Principal investigator
POC: Point of care
PreHAP: Pre-hospital diagnosis and triage of heart attack patients trial
STEMI: ST-elevation myocardial infarction
UAP: Unstable angina pectoris
