# Supplementary material for: A multicentre, randomized, controlled open-label trial to compare an Accelerated Rule-Out protocol using combined prehospital copeptin and in-hospital high sensitive troponin with standard rule-out in patients suspected of acute Myocardial Infarction – the AROMI trial
Source: Trials. 2018 Dec 12;19:683. doi: 10.1186/s13063-018-2990-z (PMC6291993; doi:10.1186/s13063-018-2990-z)
Supplement: Supplementary file 2 — AROMI biobank for future use. (DOCX 22 kb) [file 13063_2018_2990_MOESM2_ESM.docx]

AROMI Biobank for future use

Two separate blood samples from each participant are collected and stored for future use.

After copeptin analysis, the remaining plasma of the prehospital and the first in-hospital blood sample is frozen down at -80 degrees Celsius for later use.

| AROMI BIOBANK specifications | |
| --- | --- |
| Number of samples | 0-9600 (depending on number of positive consents & volume in collected samples) |
| Number of individuals | 0-4800 (depending on number of positive consents) |
| Sample type | Blood plasma |
| Sample volume | 0-2 mL (depending on volume in collected samples) |
| Storage media | Screw Cap Micro Tube, 2 ml, PP, with skirted base |
| Storage | Stored at -80 degrees Celcius |
| Sample 1 | Sample drawn in ambulance |
| Sample 2 | Sample drawn at arrival to hospital |
