# Supplementary material for: A multicentre, randomized, controlled open-label trial to compare an Accelerated Rule-Out protocol using combined prehospital copeptin and in-hospital high sensitive troponin with standard rule-out in patients suspected of acute Myocardial Infarction – the AROMI trial
Source: Trials. 2018 Dec 12;19:683. doi: 10.1186/s13063-018-2990-z (PMC6291993; doi:10.1186/s13063-018-2990-z)
Supplement: Supplementary file 3 — Data variables and registries. Description of the data variables collected in the study and the registries from which some of the variables are retrieved. (DOCX 29 kb) [file 13063_2018_2990_MOESM3_ESM.docx]

Collected data variables and data sources

Outcome data

LOS: derived from time of admission and time of discharge (see Timing)

MACE-events: See “**Adjudication of events” in protocol and Additional file 4.**

Baseline characteristics:

Age: Retrieved from the CPR-register (Danish Civil Registration System (In Danish: Central Person Register))

Gender: Retrieved from the CPR-register

Height: Retrieved from the medical record; if missing complemented from the study-CRF.*

Weight: Retrieved from the medical record; if missing complemented from the study-CRF.*

Medical history

Prior AMI: Retrieved from the study-CRF; complemented from the medical record and NPR (Danish National Patient Registry (In Danish: Landspatientregisteret)).

Existing angina: Retrieved from the study-CRF; complemented from the medical record and NPR.

Prior PCI: Retrieved from the study-CRF; complemented from the medical record and NPR.

Prior CABG: Retrieved from the study-CRF; complemented from the medical record and NPR.

Prior CAG without intervention: Retrieved from the study-CRF; complemented from the medical record and NPR.

Risk factors of ischaemic heart disease (IHD)

Hypercholesterolemia (treated or non-treated): Retrieved from the study-CRF; complemented from the medical record and NPR.

Smoking (actual or prior): Retrieved from the study-CRF; if missing complemented from the medical record.

Family disposition: Retrieved from the study-CRF; if missing complemented from the medical record.

Hypertension (treated or non-treated): Retrieved from the study-CRF; complemented from the medical record and NPR.

Diabetes (Type 1/ type 2): Retrieved from the study-CRF; complemented from the medical record and NPR.

Comorbidity

Chronic heart failure: Retrieved from the study-CRF; complemented from the medical record and NPR.

Atrial fibrillation: Retrieved from the study-CRF; complemented from the medical record and NPR.

Valvular heart disease: Retrieved from the study-CRF; complemented from the medical record and NPR.

Other cardiac comorbidity, incl. prior cardiac surgery: Retrieved from the study-CRF; complemented from the medical record and NPR.

Peripheral artery disease: Retrieved from the study-CRF; complemented from the medical record and NPR.

Carotid stenosis: Retrieved from the study-CRF; complemented from the medical record and NPR.

Prior cerebral stroke: Retrieved from the study-CRF; complemented from the medical record and NPR.

Chronic kidney failure: Retrieved from the study-CRF; complemented from the medical record and NPR.

Chronic obstructive pulmonary disease: Retrieved from the study-CRF; complemented from the medical record and NPR.

Asthma: Retrieved from the study-CRF; complemented from the medical record and NPR.

Upper gastrointestinal disease: Retrieved from the study-CRF; complemented from the medical record and NPR.

Medication

Anti-hypertensive treatment: Retrieved from the study-CRF; if missing complemented from the medical record.

Anti-platelet treatment: Retrieved from the study-CRF; if missing complemented from the medical record.

Anticoagulant treatment: Retrieved from the study-CRF; if missing complemented from the medical record.

Anti-lipid treatment: Retrieved from the study-CRF; if missing complemented from the medical record.

Anti-diabetic treatment: Retrieved from the study-CRF; if missing complemented from the medical record.

Anti-reflux/antacid treatment: Retrieved from the study-CRF; if missing complemented from the medical record.

Anti-angina treatment: Retrieved from the study-CRF; if missing complemented from the medical record.

Anti-arrhythmic treatment: Retrieved from the study-CRF; if missing complemented from the medical record.

Vital signs

Systolic blood pressure: Retrieved from the medical record; if missing complemented from the study-CRF.*

Diastolic blood pressure: Retrieved from the medical record; if missing complemented from the study-CRF.*

Heart rate: Retrieved from the medical record; if missing complemented from the study-CRF.*

Respiratory rate: Retrieved from the medical record; if missing complemented from the study-CRF.*

Temperature: Retrieved from the medical record; if missing complemented from the study-CRF.*

Oxygen saturation: Retrieved from the medical record; if missing complemented from the study-CRF.*

Oxygen treatment (Yes/No): Retrieved from the study-CRF; if missing complemented from the medical record

Symptoms

Type/character (Angina/ Atypical chest pain/Dyspnoea/Stomach pain/Cardiac arrest/ Angina within 48 h/ Other): Retrieved from the study-CRF; if missing complemented from the medical record

ECG

ST-segment elevation: Retrieved from the study-CRF; if missing complemented from the medical record

ST-segment depression: Retrieved from the study-CRF; if missing complemented from the medical record

Left Bundle Branch Block: Retrieved from the study-CRF; if missing complemented from the medical record

Right Bundle Branch Block: Retrieved from the study-CRF; if missing complemented from the medical record

Inverted T-waves: Retrieved from the study-CRF; if missing complemented from the medical record

Left ventricular hypertrophy: Retrieved from the study-CRF; if missing complemented from the medical record

Attending doctor’s clinical evaluation (serious condition/ not serious condition): Retrieved from the study-CRF

Timing

Time/date of onset of symptoms: Retrieved from the study-CRF; if missing complemented from the medical record

Time/date of maximum of symptoms: Retrieved from the study-CRF; if missing complemented from the medical record

Time/date of blood sampling: Retrieved from LABKA

Time/date of admission: Retrieved from the patient administrative system, the medical record and NPR*

Time/date of discharge: Retrieved from the patient administrative system, the medical record and NPR *

Time from onset of symptoms to blood sampling/admission/diagnosis: derived from time of onset of symptoms and time of blood sampling

Time from maximum of symptoms to blood sampling/admission/diagnosis: derived from time of maximum of symptoms and time of blood sampling

LOS: derived from time of admission and time of discharge

Presumed MACE during admission: Retrieved from the study-CRF

Diagnostic procedures during admission

ECG: Retrieved from the study-CRF, the patient administrative system, and the medical record

Chest X-ray: Retrieved from the study-CRF, the patient administrative system, and the medical record

Echocardiography: Retrieved from the study-CRF, the patient administrative system, and the medical record

Other imaging: Retrieved from the study-CRF, the patient administrative system, and the medical record

Biochemistry

Copeptin: pre- and in-hospital: Retrieved from LABKA

Hs-cTnT: pre- and in-hospital: Retrieved from LABKA

* To ensure the validity of electronically extracted data from the patient administrative system and the medical record we compared the data collected manually (via study-CRF) and the electronically extracted data for the following variables: *Height, Weight, Systolic blood pressure, Diastolic blood pressure, Heart rate, Respiratory rate, Temperature, Oxygen saturation, Oxygen treatment, Time/date of admission, Time/date of discharge*

Justified on the below presented comparison, the extracted data from the patient administrative system and the medical record is used as main source for the mentioned variables.

| Comparison of data on “Time/date of admission” & “Time/date of discharge”  Compares manually collected data and data from patient administrative system and the medical record | | | |
| --- | --- | --- | --- |
| Randomization group | Subgroup | Obs (n) | Difference in mean LOS* in hours |
| Standard | All patients | 1,050 | 0.2 |
| Standard | Early discharge** | 574 | 0.2 |
|  |  |  |  |
| Accelerated | All patients | 1,046 | 0.2 |
| Accelerated | Early discharge** | 556 | 0.0 |

* Length of stay
** discharged within 12 hours of admission. (see Endpoints )

| Comparison of data on: *Height, Weight, Systolic blood pressure, Diastolic blood pressure, Heart rate, Respiratory rate, Temperature, Oxygen saturation & Oxygen treatment.* | | | |
| --- | --- | --- | --- |
| Variable | Obs | Mean ”study-CRF” | Mean ”medical record” |
| Diastolic blood pressure | 756 | 77.8 | 77.5 |
| Systolic blood pressure | 780 | 135.0 | 135.0 |
| Temperature | 1,419 | 37.0 | 37.0 |
| Height | 1,060 | 172.8 | 172.5 |
| Weight | 1,108 | 82.0 | 82.2 |
| Heart rate | 1,529 | 73.6 | 73.6 |
| Respiratory rate | 1,526 | 16.4 | 16.4 |
| Oxygen saturation | 1,502 | 96,9 | 96,8 |
| Oxygen treatment | 930 | 11.3 (% of patients) | 11.2 (% of patients) |

Registries used in data collection

# Danish National Patient Registry (Landspatientregisteret, NPR):

Description: “The Danish National Patient Registry was established in 1976 and contains data regarding Danish patients’ hospital contacts in Denmark.”[13]

The registry is national and governmentally controlled.

Data retrieved: Admission data including admission and discharge dates and times, discharge diagnoses, admission type (acute or planned), discharge type (home or to other department).

Diagnoses given prior to and after randomization

# Danish Civil Registration System (The CPR-register)

Description: The Danish Civil Registration System is a national, governmental registry, established in 1968 “with the main purpose of providing all resident of Denmark with a personal identification number. Moreover has the purpose of registering basal person information, such as residential address”.[14] Moreover, name, birth information, nationality, kinship, marital status and vital status.

Data retrieved: Vital status including status date. Registrations of emigration or disappearance, including date of registration.

# DANARREST

Description: “DANARREST is a clinical, quality-database, which monitors and contributes to develop the quality of treatment of cardiac arrests in Danish hospitals. All cases of in-hospital cardiac arrest must be reported to the database”[15, 16]

Data retrieved: Data on date and time of suspected cardiac arrest and registered heart rhythms and defibrillations.

# Western Denmark Heart Registry

Description: The Western Denmark Heart Registry is a regional, clinical, quality-database monitoring invasive cardiac treatment and cardiothoracic surgery.

Data retrieved: Data on date and time of CAG, PCI, and CABG; data on number of treated lesions/arteries.

# The Register of Causes of Death (Dødsårsagsregisteret)

Description: The Register of Causes of Death was established in 1973 and contains information on the time of death, the causes of death, the age at the time of death, the manner and place of death. It is based on death certificates reported by a doctor. The register is administered by the Danish Health Authority.
