# Supplementary material for: A multicentre, randomized, controlled open-label trial to compare an Accelerated Rule-Out protocol using combined prehospital copeptin and in-hospital high sensitive troponin with standard rule-out in patients suspected of acute Myocardial Infarction – the AROMI trial
Source: Trials. 2018 Dec 12;19:683. doi: 10.1186/s13063-018-2990-z (PMC6291993; doi:10.1186/s13063-018-2990-z)
Supplement: Supplementary file 4 — Endpoint committee. Description of the endpoint committee and endpoint adjudication. (DOCX 83 kb) [file 13063_2018_2990_MOESM4_ESM.docx]

Description of Endpoint Committee and event adjudication

# Committee members

6-9 External cardiologists:

Jens Flensted Lassen, Lia Bang, Lene Holmvang, Birgit Jurlander, Lotte Saaby, Hans Mickley, Martin Busk

# Tasks

The Committee shall evaluate by agreement included patients in order to:

Adjudicate MACE endpoints by evaluation of patients with suspected MACE-events as assessed from: diagnoses from the Danish National Patient Registry, registrations in the Western Danish Heart database, registrations in Danarrest, registrations in LABKA and manual registrations from the index hospitalization.

In the case of ACS/AMI endpoint: define MI type (Type 1 infarction, type 2 infarction, other type of infarction or UAP, and subdivide in STEMI and NSTEMI).

In the case of elevated high sensitivity troponin during the index hospitalization, distinguish between AMI, according to the above, or myocardial injury.

# Remuneration

There will be remuneration of Endpoint Committee. Expenses for transportation, lodging and meeting facilities are borne by the sponsor.

# Adjudication

In adjudication, each Member shall determine and enter the adjudication in the trial system "Trial partner". Each patient must be adjudicated by a least two committee members. In case of disagreement, adjudication by third Committee member (Hans Mickley) determines the adjudication.

A subset of the suspected MACE-events is auto-adjudicated. This is the case for so-called definite endpoints, where information from above mentioned registries presents information which allows for immediate adjudication (see more details below). The use of auto-adjudication is conditioned on the approval from the Committee, including a committee review of min. 10% of auto-adjudicated events.

Each Committee member is assigned a number of patients for review. Each patient may have several suspected events and several confirmed events, however, only one event of each type.

# MACE endpoints

All-cause mortality

Survived cardiac arrest

Acute coronary syndrome

Non-scheduled coronary revascularization (PCI or CABG)

Life-threatening, treatment-demanding arrhythmias (3^rd^ degree AV-block, Ventricular tachycardia, or Ventricular fibrillation).

# Available data

All data is protected by a data processor contract.

Patient records are delivered to the individual Committee members on encrypted hard disks, along with data from the civil registration system, presenting updated information on mortality status.

On request, Committee members are forwarded additional materials such as: ECG, imaging characterizations (ECHO, PCI/KAG are already in the patient records), etc.

# Reporting of adjudication

Trial Partner is an online trial tool, in which Committee members register the adjudication of MACE events. Adjudications done by other committee members cannot be seen.

# Auto-adjudication:

-Definite endpoints, which allows for immediate adjudication.

The Endpoint Committee must review and approve minimum 10% of these auto-adjudications.

***All-cause death*** is auto-adjudicated if the patient is registered as dead (code “90”) in the Danish Civil Registration System (CRS)

***AMI*** is auto-adjudicated (Note that MI-type is manually adjudicated) in cases where the patient:

1. have PCI performed (with at least 1 treated lesion registered in the Western Danish Heart database) and at the time of PCI, is given AMI-diagnosis of PCI-operator

AND

1. have the PCI performed during or within 24 hours from a hospital admission with:

a. elevated troponin above the 99^th^ percentile cut point

AND

b. significant rise and/or fall of troponin (see below)

AND

1. the patient during this admission is given a diagnosis of AMI in the Danish National Registry of Patients.

***Survived Cardiac arrest*** is auto-adjudicated in cases where:

1. Patient survived to discharge

AND

- 1. a "shockable rhythm" AND "shock delivered " during an attempt of resuscitation are specified in DANARREST (see additional file 3).

OR

- 1. "shockable rhythm", "ventricular fibrillation”, “ventricular tachycardia” and “shock”, “defibrillation” or “DC- conversion”(all of these in all possible sub forms of spelling) is specified in the patient record during a hospitalization with the ICD-10 discharge diagnosis: DI460 – “cardiac arrest with successful resuscitation”.

***3^rd^ degree AV-block*** is auto-adjudicated in cases where:

1. a pacemaker is implanted during a hospitalization with third degree AV-block as discharge diagnosis, registered in the Danish National Patient Registry.

***Unplanned revascularization*** is auto-adjudicated in cases where the following three requirements are met:

1. PCI/CABG is performed (registered in the Western Danish Heart database with: at least 1 lesion treated in case of PCI, or "TRUE" in case CABG)

AND

1. the revascularization procedure is equivalently registered in the Danish National Patient Registry with a procedure-code corresponding to the operation type and date registered in the Western Danish Heart database.

AND

1. The revascularization procedure is carried out during an admission starting with an acute hospitalization registered in the Danish National Patient Registry.

# Suspected events

Events (non-auto-adjudicated events) are adjudicated by the endpoint committee.
The following events are assessed in the endpoint committee as ***suspected events***:

1. All events registered in the DAN-ARREST registry until confirmed event (SCA).
2. All events with an ICD-10 diagnosis of "Survived Cardiac arrest" (ICD-10: DI460) in the Danish National Patient Registry until confirmed event (SCA).
3. All events where "shockable rhythm", "ventricular fibrillation”, “ventricular tachycardia” and “shock”, “defibrillation” or “DC- conversion”(all of these in all possible sub forms) is specified in the patient record until confirmed event (SCA).
4. All troponin measurements above the 99^th^ percentile until confirmed event (AMI).
5. All events with an ICD-10 diagnosis of UAP (DI200, DI200B, DI200C) in the Danish National Patient Registry until confirmed event (ACS).
6. All events with an ICD-10 diagnosis of AMI (DI21, DI210, DI210A, DI210B, DI211A, DI211B, DI221 DI213, DI214, DI219) in the Danish National Patient Registry until confirmed event (AMI).
7. All events with an ICD-10 procedure code of PCI (KFNG **) in the Danish National Patient Registry until confirmed event (unplanned revascularization).
8. All events with an ICD-10 procedure code of CABG (KFN **) in the Danish National Patient Registry until confirmed event (unplanned revascularization).
9. All events with a PCI or CABG registrered in the Western Denmark Heart Database
10. All events with an ICD-10 diagnosis of ventricular tachycardia (I490B, I490BA) in the Danish National Patient Registry until confirmed event (Life-threatening arrhythmia).
11. All events with an ICD-10 diagnosis of ventricular fibrillation (I470A, I472A, I472B, I472D, I472, I478A) in the Danish National Patient Registry until confirmed event (Life-threatening arrhythmia).
12. All events with an ICD-10 diagnosis of 3^rd^ degree AV-Block (I442, I442A, I443A, I443) in the Danish National Patient Registry until confirmed event (Life-threatening arrhythmia).

# Manual adjudication of suspected events

All-cause death

is accepted if:

-the patient in the provided list: "t_person" (raw data from the social security register) appears with

-          the code "90" (= Inactive, dead) on the specified date

Survived cardiac arrest:

is accepted if:

1. the patient record provides information proving that a resuscitation attempt has been carried out (Basic or advanced cardio-/pulmonary resuscitation)
2. this attempt was carried out on the specified date
3. that this attempt were performed by health care professionals (paramedic, nurse or doctor)
4. the patient survived to discharge.

Not scheduled revascularizing coronary intervention (PCI or CABG)

is accepted if:

1. the patient record provides information proving that a non-scheduled coronary revascularization was performed.
2. that this intervention was carried out the specified date.

By coronary intervention is meant: CABG and PCI

By "not planned" is meant: procedures carried out under non-elective admissions, with the exception of cases where an acute hospitalization coincides with the date of an already planned intervention.

Life-threatening, treatment-demanding arrhythmias (3^rd^ degree AV-block, ventricular tachycardia or ventricular fibrillation).

is accepted if:

1. the patient record provides information proving that a 3^rd^ degree AV-block, ventricular tachycardia or ventricular fibrillation has been occurred.
2. the patient record provides information proving that the above have resulted in initiation of treatment (medical, implantation of cardiac pacemaker (temporary or permanent), cardioversion, or other electrophysiological treatment)

Readmissions with acute coronary syndrome (AMI and UAP):

- NSTEMI, Type 1 MI is accepted if:

1. the patient's laboratory results meets the criterion for AMIi
2. the journal can be found evidence of:
   1. overt ischemia or identification of an intracoronary thrombus at coronary angiography
   2. absence of ST-segment elevation iii
   3. Spontaneous myocardial infarction iv

- NSTEMI, Type 2 MI is accepted if:

1. the patient's laboratory results meets the criterion for AMI^i^
2. the patient record provides evidence of:
   1. overt ischemia ^ii^
   2. absence of ST-segment elevation ^iii^
   3. imbalance between oxygen supply and myocardial oxygen demand v

- NSTEMI, other type of MI is accepted if:

1. the patient's laboratory results meets the criterion for AMI^i^
2. the patient record provides evidence of:
   1. overt ischemia ^ii^
   2. absence of ST-segment elevation ^iii^
3. the criteria for Type 1 & Type 2 AMI is NOT fulfilled ^iv^ ^&^ ^v^

- STEMI, Type 1 MI is accepted if:

1. the patient's laboratory results meets the criterion for AMI^i^
2. the patient record provides evidence of:
   1. overt ischemia ^ii^
   2. the presence of ST-segment elevation ^iii^
   3. Spontaneous myocardial infarction ^iv^

- STEMI, Type 2 MI is accepted if:

1. the patient's laboratory results meets the criterion for AMI^i^
2. the patient record provides evidence of:
   1. overt ischemia ^ii^
   2. the presence of ST-segment elevation ^iii^
   3. imbalance between oxygen supply and myocardial oxygen demand v

- STEMI, other type of MI is accepted if:

1. the patient's laboratory results meets the criterion for AMI^i^
2. the patient record provides evidence of:
   1. overt ischemia ^ii^
   2. the presence of ST-segment elevation ^iii^
3. the criteria for Type 1 & Type 2 is NOT fulfilled ^iv^ ^&^ ^v^

- Myocardial injury (not part of endpoint) is accepted if:

1. the patient's laboratory results presents one hs-cTnT-measurement > 99^th^ percentile (with or without fulfilment of the biochemical criteria for AMI^i^)
2. the patient record provides evidence of:
   1. ABSENCE of overt ischemia ^ii^

- Unstable Angina Pectoris is accepted if:

1. the patient's laboratory results does not meet the criterion for AMI^i^
2. the patient record provides evidence of one of the following:
   1. New onset of severe angina pectoris.
   2. Angina pectoris occurring during rest or minimal physical exercise.
   3. Crescendo angina pectoris with increase in symptom frequency and duration in patients with prior existing chronic, stable angina pectoris.

^(i)^ Biochemical criteria for AMI:

Detection of a rise and/or fall in high sensitivity cardiac troponin with at least 1 value above the 99^th^ percentile (14 ng/L for hs-cTnT). Rise and fall are defined as follows:

1. if the lowest troponin value in question is ≤ the 99^th^ percentile, then troponin is required to rise to or fall from a value > the 99th percentile+ 50% of the 99th percentile (21ng/L for hs-cTnT).

2. if the lowest troponin value in question is between the 99th percentile and 150% of the 99th percentile (15-21 ng/L for hs-cTnT) an increase or decrease (delta) of minimum 50% of the lowest value is required (8-11 ng/L for hs-cTnT).

3. if the lowest troponin value in question is > 150% of the 99th percentile (21 ng/L for hs-cTnT) an increase or decrease (delta) of minimum 50% of the lowest value is required (11 ng/L for hs-cTnT).


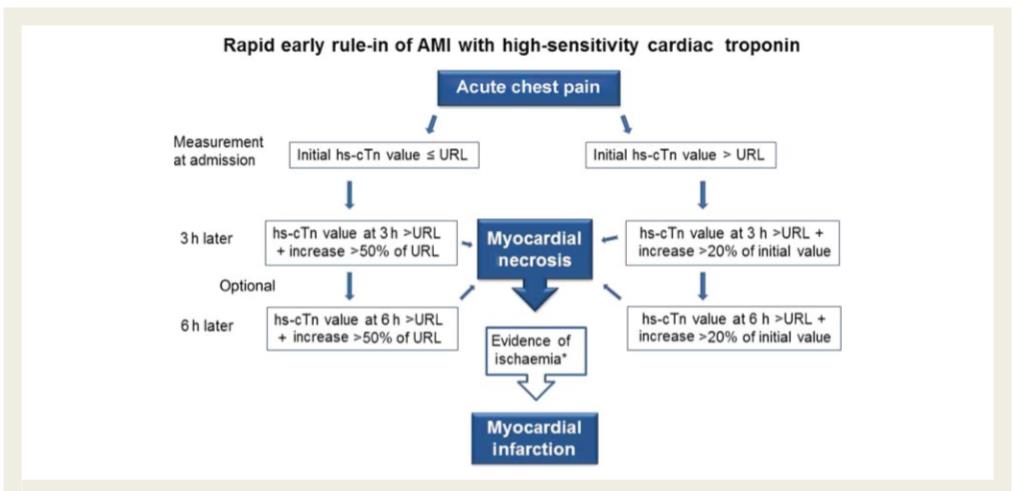


(ii) Overt ischemia:
Symptoms of ischemia. New or presumed new ischemic ECG changes. Imaging evidence of new loss of viable myocardium or new regional wall motion abnormality.

(iii) ST-elevation:
is accepted if the ECG in the patient record is described with relevant ECG changes: New ST elevation at the J point in two contiguous leads with the cut-points: ≥0.1 mV in all leads other than leads V2–V3 where the following cut points apply: ≥ 0.2 mV in men ≥ 40 years; ≥ 0.25 mV in men < 40 years; ≥0.15 mV in women or a new (or presumed new) left bundle branch block (LBBB).

(iv) Spontaneous myocardial infarction:
Atherosclerotic plaque rupture, ulceration, ﬁssuring, erosion, or dissection with resulting intraluminal thrombus in one or more of the coronary arteries leading to decreased myocardial blood ﬂow or distal platelet emboli with ensuing myocyte necrosis.

(v) Imbalance between oxygen supply and myocardial oxygen demand:

**Reduced myocardial oxygen supply**:

-Anaemia defined as haemoglobin concentration < 5.5 mmol/L for men and < 5.0 mmol/L for women;

-Shock defined as a systolic blood pressure <90 mmHg along with signs of organ dysfunction (e.g., metabolic acidosis, arterial oxygen tension < 8 kPa, oliguria [diuresis < 30 mL/hour in minimum 3 hours], or encephalopathy);

-Brady-arrhythmias needing medical treatment or pacing;

-Coronary spasm or spontaneous coronary dissection without plaque rupture.

-Coronary embolus along with an condition with increased risk of embolism (left-sided endocarditis, intra-cardiac mural thrombosis, documented venous thrombosis and a patent Foramen Ovale or atrial septum defect); or

-Respiratory failure with an arterial oxygen tension < 8 kPa and clinical signs of acute respiratory failure in ≥ 20 minutes.

**Increased oxygen demand myocardial**:

-Ventricular tachyarrhythmia with duration ≥ 20 minutes;

-Supraventricular tachyarrhythmia with a ventricular frequency > 150 strokes/min and duration ≥ 20 minutes;

-Hypertensive pulmonary oedema, defined as the presence of systolic blood pressure > 160 mmHg, signs of pulmonary oedema and the need for treatment with nitrates or diuretics; or

-Arterial hypertension with systolic blood pressure > 160 mmHg and accompanying left ventricular hypertrophy identified on Echocardiography or ECG.
