# Supplementary material for: A multicentre, randomized, controlled open-label trial to compare an Accelerated Rule-Out protocol using combined prehospital copeptin and in-hospital high sensitive troponin with standard rule-out in patients suspected of acute Myocardial Infarction – the AROMI trial
Source: Trials. 2018 Dec 12;19:683. doi: 10.1186/s13063-018-2990-z (PMC6291993; doi:10.1186/s13063-018-2990-z)
Supplement: Supplementary file 5 — Data monitoring committee. Description of the data monitoring committee and data monitoring. (DOCX 22 kb) [file 13063_2018_2990_MOESM5_ESM.docx]

Data monitoring committee

Role

The DMC will perform interim analyses to evaluate safety endpoints and study progression.

Composition of DMC

The DMC consists of two independent (sponsor and competing interests) cardiologists and a biostatistician.
Cardiologists: Rikke Sørensen and Charlotte Kragelund
Biostatistician: Morten Madsen

Interim analyses:

The committee meets after enrolment of 500, 1000 and 3000 patients in order to:

1. Evaluate safety endpoints, using data extractions from the Danish National Registry of Patients.
2. Evaluation of study endpoints

Safety endpoints

1. 30 day rate of combined MACE, occurring after discharge or during index admission (in total)
2. 30 day rate of combined MACE, occurring during index admission.
3. 30 day rate of combined MACE, occurring after discharge.
4. 30 day rate of MACE (separately)
5. 90 and 365 day rate of combined MACE, occurring after discharge; under index admission; and in total (separately and combined MACE)

MACE: see **Outcome Measures.**

Study endpoints: see **Outcome Measures.**

Stopping guidelines

The DMC shall recommend termination of the study if MACE rate is shown to be significantly higher in the intervention group (*“Accelerated”*) compared with the control group (*“Standard”*), with a p-value below 0.01.

This applies to safety endpoint:

1. 30 day rate of combined MACE, occurring after discharge or during index admission (in total)
2. For the safety endpoints 2-5 applies that:

- if MACE rate is shown to be significantly higher in the intervention group (*“Accelerated”*) compared with the control group (*“Standard”*), with a p-value below 0.01, the DMC shall inform the sponsor/project management. The consequence of this depends on the remaining data and is determined in agreement between DMC and sponsor/project management.

1. For all 5 safety endpoints applies that:

- if MACE rate is shown to be significantly higher in the control group (*“Standard”*) compared with the intervention group (*“Accelerated”*), with a p-value below 0.01, the DMC shall inform the sponsor/project management. The consequence of this depends on the remaining data and is determined in agreement between DMC and sponsor/project management.

Other recommendations from the DMC

The total MACE rate (total for both diagnostic groups) using data extractions from the Danish National Registry of Patients, is reported to sponsor, in order to evaluate the need for prolongation of study period or increasing the number of study sites or alternatively recalculation of sample size.

Readmissions with the first 24 hours are reviewed, initially using data extractions from the Danish National Registry of Patients. If the registered discharge diagnosis (from the readmission) raises suspicion of possible connections between readmission and the study intervention (specifically early discharge based on Copeptin and hs-cTnT) medical journals are reviewed for the concerned patients. If the suspicion of connection is confirmed or maintained after journal review, the event is reported as a ” Suspected Unexpected Serious Adverse Reaction” to the sponsor/project management in order to report the event to The National Research Ethics Committee, as dictated in ”Indberetningspligten i sundhedsvidenskabelige forsøg, bortset fra lægemiddelforsøg”.

Data analysis in DMC

All data (un-blinded) are available for the DMC. The data analysis is performed by biostatistician.

Remuneration

The biostatistician is bought to participate from the Department of Clinical Epidemiology, Aarhus University Hospital, Aarhus, Denmark. The two cardiologists are participating unpaid.
Expenses for transport, meals and meetings are covered.
